# HOW MEDICARE ADVANTAGE IS SHAPING THE CARE OF OLDER ADULTS WITH SERIOUS ILLNESS

**DOI:** 10.1093/geroni/igad104.1359

**Published:** 2023-12-21

**Authors:** Claire Ankuda

**Affiliations:** Icahn School of Medicine at Mount Sinai, New York City, New York, United States

## Abstract

The Medicare Advantage (MA) program currently insures half of Medicare beneficiaries, and yet there are major gaps in our understanding of how it is impacting older adults, particularly those with serious illness and at the end of life. This population is particularly vulnerable to lapses in care quality in MA given their complex health care needs and challenges navigating MA cost-control mechanisms such as prior authorizations and narrow networks. I will present a framework to consider how MA is influencing care delivery and outcomes for older adults, a review of what we know about the impact of the dramatic growth of MA plans, and emerging data on the experience of older adults with serious illness in MA. This session will highlight best-practices regarding use of claims and population data to evaluate the MA program.

